# Microbiota–gut–brain axis in neurodegenerative diseases: molecular mechanisms and therapeutic targets

**DOI:** 10.1186/s43556-025-00307-1

**Published:** 2025-09-15

**Authors:** Ce Chen, Guo-qing Wang, Dai-di Li, Feng Zhang

**Affiliations:** 1 Key Laboratory of Basic Pharmacology of Ministry of Education and Joint. International Research Laboratory of Ethnomedicine of Ministry of Education and Key Laboratory of Basic Pharmacology of Guizhou Province and Laboratory Animal Centre, Zunyi Medical University, Zunyi, Guizhou, China; 2 State Key Laboratory of Natural Medicines, School of Traditional Chinese Medicines, China Pharmaceutical University, Nanjing, Jiangsu China; 3 Department of Pharmacy, The First Affiliated Hospital of Bengbu Medical University, Bengbu Medical University, Bengbu, Anhui China

**Keywords:** Neurodegenerative diseases, Gut microbiota, Gut-brain-axis, Metabolite, Neuroinflammation

## Abstract

The microbiota–gut–brain axis (MGBA) is an intricate bidirectional communication network that links intestinal microbiota with the central nervous system (CNS) through immune, neural, endocrine, and metabolic pathways. Emerging evidence suggests that dysregulation of the MGBA plays pivotal roles in the onset and progression of neurodegenerative diseases. This review outlines the key molecular mechanisms by which gut microbes modulate neuroinflammation, blood–brain barrier integrity, protein misfolding, and neuronal homeostasis. We discuss how microbial metabolites, such as short-chain fatty acids, tryptophan derivatives, and bile acids, interact with host to influence CNS functions. Disease-specific features are described across Alzheimer’s disease, Parkinson’s disease, Multiple sclerosis, and Amyotrophic lateral sclerosis, emphasizing the distinct and overlapping pathways through which gut dysbiosis may contribute to pathogenesis. We further explore the translational potential of microbiota-targeted therapies, including probiotics, fecal microbiota transplantation, dietary interventions, and small-molecule modulators. While preclinical results are promising, clinical trials reveal considerable variability, highlighting the need for personalized approaches and robust biomarkers. Challenges remain in deciphering causal relationships, accounting for inter-individual variability, and ensuring reproducibility in therapeutic outcomes. Future research should integrate multi-omics strategies, longitudinal human cohorts, and mechanistic models to clarify the role of the MGBA in neurodegeneration. Collectively, understanding the MGBA provides a transformative perspective on neurodegenerative disease mechanisms and offers innovative therapeutic avenues that bridge neurology, microbiology, and precision medicine.

## Introduction

Neurodegenerative diseases (NDDs) – including Alzheimer’s disease (AD), Parkinson’s disease (PD), Huntington’s disease (HD), amyotrophic lateral sclerosis (ALS), and others – are characterized by progressive loss of neurons leading to cognitive or motor deficits [1]. These conditions impose a tremendous burden, affecting tens of millions worldwide as populations age [2]. Traditionally, NDD pathogenesis has been viewed through a neurocentric lens focusing on protein misfolding, synaptic dysfunction, and central immune activation [3]. However, mounting evidence points to an intimate connection between the brain and the gastrointestinal tract in these disorders [4]. Patients with NDDs frequently exhibit gastrointestinal disturbances or microbiome alterations years before classic neurological symptoms emerge [5, 6]. For example, chronic constipation can precede PD motor Symptoms by up to 20 years, and many AD patients show distinct gut microbiota profiles compared to healthy peers [7]. Such observations suggest that perturbations in the microbiota–gut–brain axis (MGBA) may play a role in disease initiation or progression.

The MGBA refers to the bidirectional communication network linking the gut’s resident microbiota and the central nervous system (CNS) [8–10]. Through neural, immune, endocrine, and metabolic signaling pathways, the gut microbiome can influence brain physiology, while the brain can in turn modulate gut microbial composition via stress hormones and autonomic innervation [11, 12]. Crucially, this cross-talk is a two-way street: CNS pathology or stress can alter gut function and microbiota, potentially creating a vicious cycle [13]. For instance, psychological stress activates the hypothalamic–pituitary–adrenal (HPA) axis and sympathetic nerves, leading to cortisol and catecholamine release that increases intestinal permeability and disrupts the gut habitat [14, 15]. The resulting leakage of microbial molecules (e.g. endotoxin) can trigger systemic inflammation that further exacerbates neuroinflammation, illustrating how brain disorders are not confined to the CNS but involve a systemic network including the gut ecosystem [16].

In this review, we synthesize current knowledge of how gut microbes and their metabolites interact with the host to influence neurodegenerative processes. We begin by outlining the components of the MGBA and its major communication pathways. Next, we detail several mechanistic links by which the microbiome can trigger or protect against neurodegeneration – spanning immune modulation, metabolic and neuroendocrine signaling, microbial neurotransmitter production, and effects on protein aggregation and epigenetic regulation. We then examine four representative NDDs (AD, PD, ALS, and MS), highlighting disease-specific gut microbiome alterations and MGBA-related mechanisms identified in each. (Although multiple sclerosis (MS) is classically an autoimmune inflammatory demyelinating disease rather than a primary proteinopathy, we include it here due to overlapping chronic CNS injury and immune dysregulation influenced by the microbiome.) For each disease, specific microbial taxa, metabolites, and pathways implicated in pathogenesis are discussed. Finally, we explore therapeutic implications: strategies to restore a healthy microbiome or modulate MGBA signals – from diets and probiotics to fecal microbiota transplantation (FMT) and metabolite-based interventions. We also summarize emerging biomarkers and ongoing clinical trials, and consider challenges such as inter-individual variability and the need to establish causal relationships. By integrating these insights, we aim to demonstrate how targeting the MGBA provides novel multi-targeted opportunities to understand and combat neurodegenerative diseases.

## Components and communication pathways of the microbiota–gut–brain axis

### MGBA components

The MGBA is a complex, integrated system spanning the gut and brain. Central to this axis is the gut microbiota – the trillions of commensal microorganisms (bacteria, viruses, archaea, fungi) that reside primarily in the colon [17]. The intestinal mucosa forms a critical interface between these microbes and the host: a single-cell epithelial layer with tight junctions that limit bacterial translocation, overlain by mucus and patrolled by immune cells [18]. Specialized enteroendocrine cells in the gut lining detect luminal contents and release neuroactive hormones, while gut-associated lymphoid tissue (GALT) coordinates immune responses to microbes. Immediately beneath the epithelium, mucosal immune cells (dendritic cells, lymphocytes) continuously sample microbial antigens and can become activated [19]. Once activated, these cells and their cytokines circulate systemically, including to the brain, thereby linking gut immunity to CNS homeostasis.

**Fig. 1:**
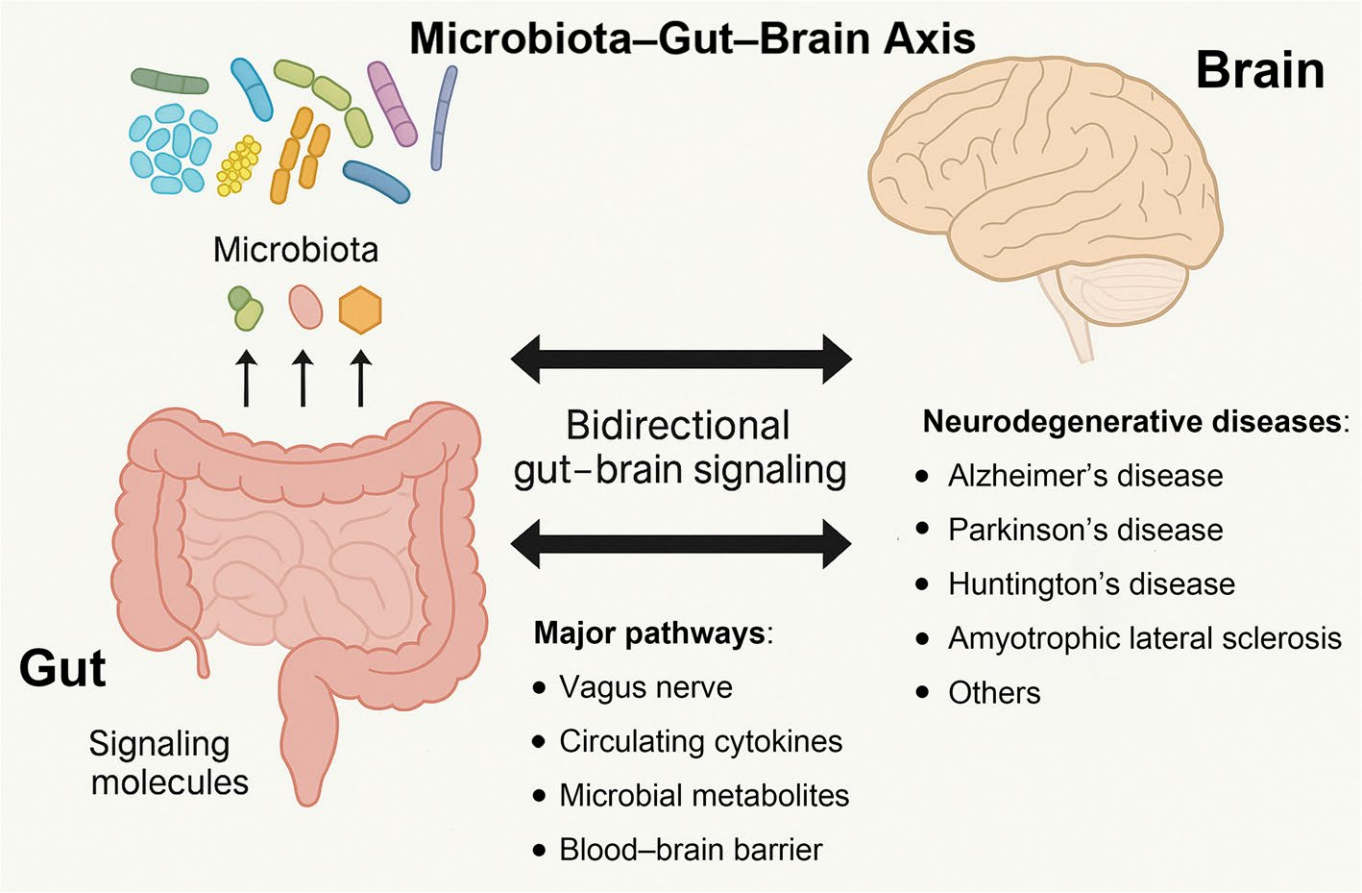
Schematic illustration of the microbiota–gut–brain axis (MGBA). This bidirectional communication network integrates microbial, neural, immune, and endocrine signals between the gastrointestinal tract and central nervous system. Major pathways include the vagus nerve, circulating cytokines, microbial metabolites (e.g., short-chain fatty acids), and modulation of the blood–brain barrier (BBB). The MGBA influences neurodevelopment, immune activation, neuroinflammation, and neurotransmission

Another key component is the enteric nervous system (ENS) – an extensive network of ~ 500 million neurons embedded in the gut wall (sometimes termed the “second brain”) [20]. The ENS regulates gut motility, secretion, and blood flow and communicates bidirectionally with the central autonomic circuits via the vagus nerve and sympathetic pathways [20]. The vagus nerve is especially important, providing a direct neural highway between gut and brainstem: vagal afferent fibers transmit sensory signals from intestinal receptors, while efferent fibers carry brain commands to influence gut activity [21]. Additional sympathetic and spinal afferent nerves connect the gut to the spinal cord, conveying visceral pain or discomfort and modulating gut immune activity [22]. In the brain, the HPA axis represents a neuroendocrine arm of the MGBA; it translates stress signals into systemic hormone release (e.g. cortisol) that can alter gut barrier integrity and immune function [23]. Brain structures such as the blood–brain barrier (BBB) and resident microglia also partake in the MGBA, as they respond to circulating microbial metabolites and inflammatory mediators; notably, BBB permeability determines which gut-derived factors can access the CNS parenchyma [24]. In summary, the MGBA comprises: (i) the gut microbiota; (ii) the intestinal barrier and mucosal immune system; (iii) circulating immune cells and cytokines; (iv) the ENS and vagus nerve connecting to (v) central autonomic circuits and HPA stress pathways; and (vi) CNS interfaces (BBB, microglia, etc.) that sense peripheral signals (as shown in Fig. 1). Disruption of any one component (for example, gut dysbiosis or a “leaky” gut lining) can reverberate throughout this interconnected system.

### Communication pathways

**Fig. 2:**
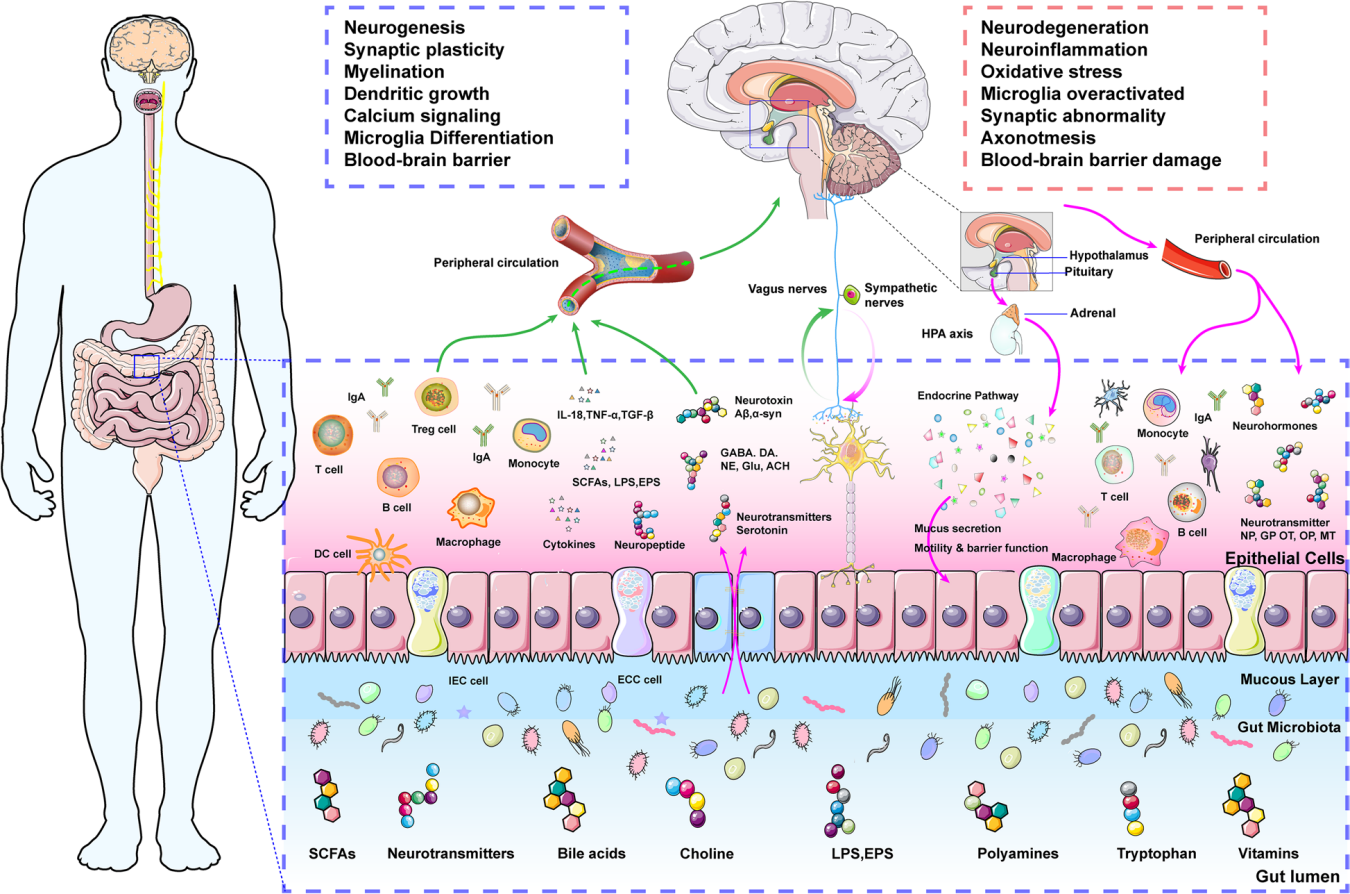
Bidirectional Communication Between the Gut Microbiota and the Brain. The communication between the gut microbiota and the brain is bidirectional and involves complex interactions across the nervous, immune, and endocrine systems, mediated by microbial metabolites. The gut microbiota serves as a critical biological foundation for these interactions, influencing brain function via pathways such as the vagus nerve, ENS, neurotransmitter release, and the regulation of neuroactive metabolites. Immune system modulation occurs through cytokines, while neuroendocrine regulation is mediated by intestinal epithelial cells (IECs) and the hypothalamic–pituitary–adrenal (HPA) axis. Dysbiosis, characterized by the depletion of beneficial metabolites, the accumulation of toxic metabolites, and the imbalance of pathogens, disrupts these pathways, impairing the blood–brain barrier (BBB) and immune function, which contributes to the initiation and progression of neurological disorders

**Fig. 3:**
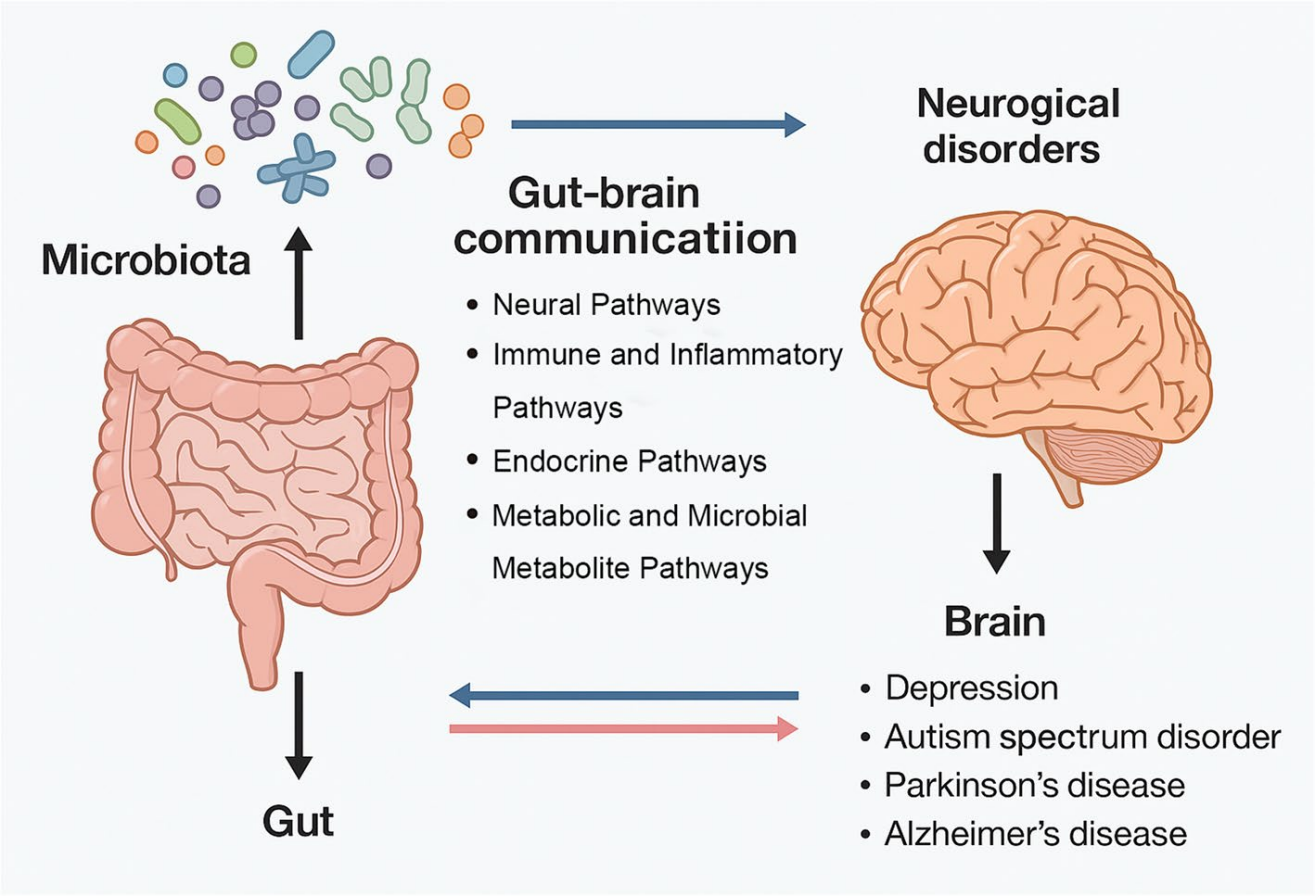
Disease-specific alterations in the MGBA across major neurodegenerative disorders. Distinct profiles of microbial composition and metabolite production have been reported in Alzheimer’s disease (AD), Parkinson’s disease (PD), multiple sclerosis (MS), and amyotrophic lateral sclerosis (ALS), influencing protein aggregation, immune dysregulation, and neurodegeneration through divergent mechanisms

Multiple interdependent signaling routes mediate cross-talk along the MGBA. Four broad categories are classically described (as shown in Fig. 2–3) [25, 26]:

#### Neural pathways

Sensory neurons and nerves relay signals between gut and brain [27]. Chief among these is the vagus nerve, which rapidly conveys information about gut state to the brainstem and vice versa. Vagal afferents detect mechanical stretch, nutrients, and microbial molecules in the gut, triggering brainstem nuclei that influence mood, appetite, and parasympathetic output. Vagal efferents, in turn, modulate gastrointestinal secretion, motility, and even local immune responses [28]. Certain gut bacteria can directly stimulate vagal pathways by producing neurotransmitters or neuromodulators. For example, microbial metabolites such as γ-aminobutyric acid (GABA), serotonin (5-HT), and histamine can activate vagal afferent endings or ENS neurons [29, 30]. This provides a route for microbial byproducts to influence brain activity in real time. A striking illustration of gut–brain neural connectivity is seen in PD: misfolded α-synuclein protein aggregates, a hallmark of PD, are hypothesized to originate in the gut and spread to the brain via vagal nerve fibers in a prion-like fashion [31]. Supporting this, individuals who underwent early-life vagotomy (surgical cutting of the vagus) have a lower subsequent risk of developing PD [32, 33]. Aside from the vagus, sympathetic fibers and spinal afferents also participate, transmitting visceral pain signals and regulating gut immune and mucus responses [32, 33]. Through these neural circuits, the gut can influence brainstem and limbic activity (affecting mood, stress responses, etc.), while brain states (e.g. stress) can alter gut motility and secretion.

#### Immune and inflammatory pathways

Gut microbes profoundly shape the host immune system from development through adulthood. Beneficial commensals generally promote immune tolerance and help reinforce the intestinal barrier, whereas an overgrowth of pathogenic bacteria or loss of key symbionts (dysbiosis) can provoke systemic inflammation [34]. Microbial-associated molecular patterns (MAMPs) such as lipopolysaccharide (LPS) from Gram-negative bacteria can breach a compromised gut barrier and enter circulation, where they activate Toll-like receptors (e.g. TLR4) and other innate immune sensors in peripheral tissues and the brain [35, 36]. Even low-grade leakage of endotoxin (LPS) from the gut can trigger chronic neuroinflammation: LPS in the bloodstream has been shown to activate microglia in the brain via TLR4/NF-κB signaling, thereby contributing to neuronal injury [37]. In parallel, gut-resident T cells conditioned by the microbiota (for instance, pro-inflammatory Th17 cells versus anti-inflammatory regulatory T cells) can traffic to the CNS [38]. Certain gut bacteria drive Th17 cell expansion; in mouse models of MS, colonization with specific segmented filamentous bacteria induces Th17 cells that infiltrate the CNS and worsen inflammation [39]. Conversely, short-chain fatty acids (SCFAs) produced by fiber-fermenting bacteria foster regulatory T cells (Tregs) that secrete anti-inflammatory cytokines like IL-10 [40]. In an experimental autoimmune encephalomyelitis (EAE) model of MS, a high-fiber diet that boosts SCFA production expanded Foxp3^+^ Tregs, strengthened the gut barrier, and reduced CNS inflammation and disease severity [41]. Immune signaling along the MGBA is bidirectional: CNS stress and inflammation can alter gut immunity via neuroendocrine pathways (e.g. stress-induced corticosteroids suppress gut immune responses), creating feedback loops between psychological stress and gut inflammation [42]. Overall, immune-mediated communication allows gut microbes to influence systemic and brain inflammation, and likewise permits CNS perturbations to affect intestinal immune homeostasis.

#### Endocrine and metabolic pathways

The gut is often termed the body’s largest endocrine organ. Enteroendocrine cells distributed along the intestinal lining sense luminal nutrients and microbial metabolites and release hormones and neuropeptides that act both locally and systemically. For example, peptide YY and glucagon-like peptide-1 (GLP-1) are released in response to food intake and influence appetite and glucose metabolism, with receptors in the brain that affect satiety and cognitive function [43]. The gut microbiota modulates levels of these hormones; SCFAs produced by bacterial fermentation of dietary fiber stimulate colonic cells to secrete peptide YY and GLP-1, which signal the brain to regulate appetite and insulin sensitivity [44]. Another example is serotonin: around 90% of the body’s serotonin is produced in the gut by enterochromaffin cells, and certain commensal bacteria (especially spore-forming Firmicutes) have been Shown to promote intestinal 5-HT biosynthesis [45]. Thus, microbial activity can influence neurotransmitter levels that modulate mood and cognition. The HPA axis also integrates into this network: gut microbes can affect cortisol dynamics by influencing the host’s metabolism of tryptophan into metabolites like kynurenine that impact HPA feedback loops [46, 47]. Additionally, microbes produce or consume numerous metabolites – amino acids, bile acids, choline derivatives, vitamins – which can enter circulation and act on distant organs including the brain [48]. For instance, certain gut bacteria metabolize dietary choline into trimethylamine (TMA), which the host then converts to trimethylamine N-oxide (TMAO); TMAO has been implicated in promoting inflammation and has been associated with increased risk of stroke and cognitive impairment [44, 49]. Conversely, some microbial metabolites are neuroprotective: the bacterial production of vitamins (like certain B vitamins) in the gut can support neuronal health [50]. In summary, through a vast array of chemically diverse compounds, the microbiome exerts endocrine-like effects on the host, influencing metabolic status, stress reactivity, and even synaptic plasticity.

#### Microbial neurotransmitters and neuromodulators

Beyond influencing host metabolite and hormone levels, gut microbes themselves produce numerous small molecules that can directly affect neuronal function. These include classical neurotransmitters (GABA, serotonin, dopamine), short-chain fatty acids (butyrate, propionate, acetate), and other neuromodulators (e.g. tryptophan metabolites, phenolic compounds) [44]. Many of these molecules can activate receptors on the vagus nerve or cross the BBB to act in the brain. For example, microbial GABA produced in the colon may interact with enteric or vagal GABA receptors, potentially influencing anxiety-like behavior in mice [51]. Certain spore-forming gut bacteria stimulate intestinal serotonin production, which can alter signaling in the brain and has been linked to changes in mood and gastrointestinal motility [45]. Bacterial metabolites can also modulate neuroplasticity; a notable case is the production of metabolites that affect microglial maturation and function [52]. Germ-free mice (lacking microbiota) show defects in microglial development and an exaggerated neuroinflammatory response, which can be normalized by reintroducing SCFA-producing bacteria [13]. This indicates that microbial signals are required for proper CNS immune balance. Additionally, some bacterial products may influence protein aggregation processes implicated in NDDs [53]. Emerging research suggests that bacterial amyloid proteins (produced by biofilm-forming gut bacteria) might prime the host’s immune system and cross-seed misfolding of host proteins like α-synuclein or Aβ, although this remains an area of debate [54]. On the other hand, beneficial metabolites such as butyrate can activate cellular mechanisms for protein clearance: butyrate readily crosses the BBB and can inhibit histone deacetylases, thereby activating gene expression programs that enhance autophagy and reduce toxic protein aggregates [55]. Indeed, in experimental models, butyrate treatment has been shown to induce autophagy and improve clearance of misfolded proteins, as well as improve synaptic and cognitive function in mice with neurodegenerative pathology [56]. In summary, the microbiome produces a pharmacopoeia of neuroactive compounds. These microbial “chemicals” can act on the ENS and vagus or reach the brain to modulate neurotransmission, neuroinflammation, and neuronal health, representing a direct molecular link between gut bacteria and brain function.

Taken together, these interacting pathways – neural, immune, endocrine/metabolic, and microbial metabolic routes – constitute the microbiota–gut–brain communication network. They provide multiple avenues through which changes in the gut microbiome can influence central processes relevant to neurodegeneration (and vice versa). In the sections below, we delve into how disruptions in these MGBA pathways have been implicated in specific neurodegenerative diseases.

## Mechanistic links between gut dysbiosis and neurodegeneration

This section delineates the mechanistic pathways through which the microbiota–gut–brain axis influences neurodegenerative processes. We highlight immune, metabolic, and neural routes, with emphasis on converging evidence from experimental and clinical studies.

### Neuroinflammation and immune activation

Chronic inflammation is a unifying feature in many NDDs, and gut microbes are emerging as key modulators of systemic and CNS inflammatory tone. Dysbiosis (an imbalanced microbiome) can promote a pro-inflammatory state via several mechanisms [57, 58]. As described above, increased intestinal permeability (“leaky gut”) allows LPS and other pro-inflammatory microbial products to enter circulation [59]. In patients with AD and PD, higher blood levels of LPS and other endotoxins have been correlated with markers of neuroinflammation and cognitive decline [60, 61]. Experimentally, peripheral administration of LPS in animals induces microglial activation and can exacerbate amyloid pathology and neurodegeneration [62]. Even in humans, low-dose endotoxin infusion is used as a model to study immune-to-brain signaling; it causes transient mood and memory impairments accompanied by elevated inflammatory cytokines in the CNS [63]. Gut microbes also shape the pool of circulating immune cells [64]. For example, certain *Clostridia* in the gut promote the development of Foxp3^+^ Treg cells that produce IL-10 and restrain inflammation [65]. Loss of these beneficial microbes could reduce Treg abundance, tilting the immune system toward a pro-inflammatory phenotype [66]. In MS, a condition with autoimmune neuroinflammation, patients often show a microbiome signature that fosters pro-inflammatory T cells (like Th17 cells) at the expense of Tregs [67, 68]. Indeed, fecal samples from MS patients, when transplanted into germ-free mice, can exacerbate autoimmune encephalitis, whereas feces from healthy donors are less pathogenic [69]. Conversely, enriching the gut microbiota with fiber-fermenting bacteria increases SCFA production and has protective effects: SCFAs signal through receptors like GPR43/GPR109A on immune cells to suppress NF-κB activation and induce Tregs [70]. Treatment of mice with sodium butyrate (a bacterial SCFA) alleviates neuroinflammation in models of AD and MS by dampening microglial activation and promoting an anti-inflammatory milieu [71]. Another immunomodulatory microbial metabolite is tryptophan-derived indoles, which activate the aryl hydrocarbon receptor (AhR) on astrocytes and intestinal immune cells [72]. Lower levels of key indole metabolites have been observed in MS patients, and their absence is linked to reduced AhR signaling and impaired gut barrier function [73]. Supplementing such metabolites or probiotic strains that produce them (e.g. certain *Lactobacillus* species) could help restore immune homeostasis [74]. In summary, gut dysbiosis may contribute to neurodegeneration by shifting the immune system toward a pro-inflammatory state, breaching the gut barrier, and chronically activating microglia and astrocytes in the brain. On the other hand, a balanced microbiota producing sufficient SCFAs, tryptophan metabolites, and other immunoregulatory factors supports an anti-inflammatory, neuroprotective environment.

### Blood–brain barrier and metabolic homeostasis

The integrity of the BBB and the brain’s metabolic environment are influenced by the gut microbiota [49]. SCFAs play a complex role here. On one hand, SCFAs (especially butyrate) strengthen the gut barrier and have anti-inflammatory effects that indirectly protect the BBB [75]. Butyrate also can cross into the bloodstream and reach the brain, where it serves as an energy substrate for neurons and glia and as an epigenetic regulator (through inhibition of histone deacetylases) [76]. This epigenetic action tends to enhance the expression of genes involved in neurotrophic factor production, synaptic plasticity, and cellular stress resistance. In models of AD, oral butyrate administration improved BBB tight junction integrity and reduced the infiltration of peripheral immune cells into the brain [77]. Butyrate has even been shown to ameliorate cognitive deficits in AD mice when given at late stages of disease. On the other hand, certain SCFAs under specific conditions might contribute to pathology: for example, a recent study found that butyrate and propionate can activate the NLRP3 inflammasome in human macrophages under inflammatory stress, suggesting a potential pro-inflammatory role in some contexts [78, 79]. Nonetheless, overall SCFA depletion (as seen with low-fiber diets or dysbiosis) is generally associated with worse outcomes in aging and neurodegeneration due to loss of their beneficial gut and brain effects.

Beyond SCFAs, other microbial metabolites affect brain metabolism and vascular function. Gut bacteria regulate bile acid pools and composition; some microbially modified bile acids (like iso-deoxycholic acid) can cross into the brain and have been shown to modulate microglial activity and cholesterol metabolism in neurons [80]. The gut microbiota also influences circulating levels of amino acids such as glutamate and glycine, which are key neurotransmitters [81, 82]. Alterations in gut bacteria have been linked to changes in the serum metabolome in conditions like ALS and PD, including altered levels of amino acid derivatives that can affect brain excitability or mitochondrial function [83, 84]. For example, hyperactivation of the glutamate system is implicated in ALS and PD, and some gut-derived metabolites (e.g. propionate) have been found to support the astrocyte-neuron glutamate–glutamine cycle and confer neuroprotective effects [85]. Microbial production of vitamins (B vitamins, vitamin K) and antioxidants (e.g. enterolactone from polyphenols) can also influence neuronal resilience to metabolic stress [86, 87]. One intriguing recent discovery is that gut microbes can produce small amounts of ammonia and other compounds that affect brain metabolism: a 2025 study showed that manipulating the gut microbiome altered brain amino acid levels and stress susceptibility in mice, partly via microbe-derived ammonia affecting neurotransmitter cycling [88]. In summary, dysbiosis might contribute to neurodegeneration by disrupting metabolic homeostasis – reducing beneficial metabolites (SCFAs, vitamins) and increasing potentially neurotoxic ones (e.g. ammonia, TMAO) – as well as by impairing the integrity of barriers like the BBB. Conversely, maintaining a healthy microbiome supports metabolic and vascular conditions that are conducive to brain health.

### Protein misfolding and aggregation

A defining feature of many NDDs is the accumulation of misfolded, aggregation-prone proteins (Aβ and tau in AD, α-synuclein in PD, SOD1/TDP-43 in ALS, etc.) [53]. There are emerging links between the microbiome and these proteopathic processes. One hypothesis is that bacterial amyloids and other proteins might seed or accelerate aggregation of host proteins. Many gut bacteria (e.g. *E. coli*, Curli-producing bacteria) secrete amyloid-like fibers as part of biofilms [89, 90]. These bacterial amyloids can be structurally similar to neuronal amyloids and may trigger cross-seeding or prime the innate immune system in a way that makes it overreact to misfolded host proteins. In PD models, oral administration of Curli-producing bacteria enhanced α-syn aggregation and motor deficits in mice, whereas germ-free or antibiotic-treated mice had less α-syn pathology [91, 92]. Another line of evidence comes from the “prion-like” transmission of α-syn: as noted earlier, pathology may start in the gut and propagate via the vagus nerve to the brainstem [93]. Gut microbiota composition can modulate this process – for example, certain microbial metabolites might affect α-syn misfolding or clearance [94]. A study in mice showed that specific SCFAs accelerated α-syn aggregation and microglial activation, whereas germ-free mice had delayed pathology [95]. However, the role of SCFAs in protein aggregation is complex (beneficial in some contexts, possibly detrimental in others as discussed) [96]. Another important mechanism is autophagy, the cellular waste-clearance process that helps remove misfolded proteins [97]. Some microbiota-derived signals promote autophagy: butyrate can induce autophagy in neurons and glia by inhibiting HDACs and activating pro-autophagic genes [98]. Propionate has also shown neuroprotective effects via enhancing remyelination and possibly facilitating debris clearance in demyelinating disease models [99]. Moreover, gut microbes influence systemic levels of acetate, which was recently shown to be crucial for microglial phagocytosis of amyloid; germ-free or antibiotic-treated mice had impaired microglial clearance of Aβ plaques, which could be restored by supplying acetate [100]. There is also evidence that peripheral inflammation driven by gut dysbiosis can reduce expression of key protein degradation systems in the brain (such as ubiquitin–proteasome pathway and autophagy genes), thereby accelerating the accumulation of toxic proteins [101]. On a therapeutic note, some microbiota-targeted treatments have reduced protein aggregates in models: long-term broad-spectrum antibiotics reduced Aβ deposition and microglial reactivity in an AD mouse model, and recolonization with a simplified microbiota reversed some of these effects, indicating that specific microbial communities can either exacerbate or ameliorate protopathic cascades [102]. In summary, while research is still early, it appears gut microbes can influence protein misfolding disorders both indirectly (via inflammation and metabolism) and directly (via amyloid cross-seeding and modulation of protein clearance pathways). This adds yet another layer to how the MGBA can shape neurodegenerative disease trajectories.

### Epigenetic and neuronal signaling pathways

The gut microbiome can affect gene expression and signaling pathways in the brain through epigenetic modifications and receptor-mediated signaling. A prime example is histone deacetylase (HDAC) inhibition by SCFAs like butyrate [103–105]. HDAC inhibition leads to a more permissive chromatin state, enhancing transcription of genes involved in neuronal survival, synaptic plasticity, and memory formation [106]. This is one reason why butyrate is being explored as a cognitive enhancer and neuroprotective agent – it essentially acts as an epigenetic modulator derived from the microbiome. In aging rodents, butyrate administration improved learning and memory, presumably by upregulating brain-derived neurotrophic factor (BDNF) and other plasticity-related proteins [76, 107]. Another SCFA, acetate, has been shown to enter the brain and become a substrate for acetyl-CoA in neurons and glia, thereby influencing histone acetylation and energy metabolism in the CNS [108]. The microbiota also influences DNA methylation patterns via production of methyl donors and modulators (e.g. folate producers in the gut can affect host methylation capacity) [109–111]. Such epigenetic changes might impact genes related to neurodegeneration [112, 113]. For example, hyperhomocysteinemia (linked to gut microbial metabolism) can alter DNA methylation in the brain and has been associated with increased AD pathology [114].

Microbial metabolites can engage specific neuronal receptors as well. G protein-coupled receptors (GPCRs) in the brain and on peripheral nerves can respond to gut-derived ligands [115]. Niacin receptors (HCAR2) on microglia respond to butyrate and other SCFAs, triggering anti-inflammatory signaling [116]. Free fatty acid receptors (FFAR2/3) on peripheral afferents detect SCFAs and can modulate serotonin release and appetite signals [117]. TGR5 and FXR, receptors for bile acids, are expressed in brain cells and on vagal afferents; microbial alterations of bile acids can therefore influence these receptors and downstream pathways affecting glucose metabolism and inflammation in the brain [118]. Additionally, pattern recognition receptors like TLR2 and TLR4 on microglia can be chronically stimulated or desensitized by repetitive exposure to microbial MAMPs translocating from the gut, potentially affecting how microglia respond to misfolded proteins (either by over-reacting and causing bystander damage, or by entering a tolerant state that might impair clearance of aggregates) [71, 119]. Finally, gut microbes can affect neurogenesis: a fascinating study showed that fecal transplants from young mice into old mice improved neurogenesis and cognition in the old mice [120]. The effect was attributed to microbial metabolites that promoted a more youthful systemic environment (for example, boosting the production of certain short-chain fatty acids and reducing pro-inflammatory cytokines) [121]. Thus, through a combination of epigenetic reprogramming, receptor-mediated signaling, and modulation of neurotransmitter systems, the gut microbiome can influence fundamental neuronal processes like synaptic plasticity, neurogenesis, and cell survival. Disruption of these influences by dysbiosis could thereby contribute to the synaptic dysfunction and neuronal loss seen in neurodegenerative diseases.

Collectively, a dysregulated MGBA can promote neurodegeneration via multiple converging mechanisms: chronic peripheral and central inflammation, impaired barrier and metabolic support for the brain, accelerated protein misfolding, and diminished neuroprotective signaling. Conversely, maintaining or restoring a healthy microbiome may bolster the brain’s resilience by reducing inflammation, enhancing protein clearance, and providing neurotrophic signals. We next turn to evidence from specific disorders that exemplify these general principles. In summary, these mechanistic insights underscore the multifactorial nature of the MGBA, where immune activation, metabolic signaling, and neuronal communication collectively contribute to disease progression. Such complexity highlights potential nodes for therapeutic intervention.

## Microbiome alterations in specific neurodegenerative diseases

Following the delineation of mechanistic links, the next consideration is how these pathways vary across specific neurodegenerative diseases. In this section, we systematically review disease-specific microbiome alterations, with a focus on Alzheimer’s, Parkinson’s, and related disorders.

### Alzheimer’s Disease (AD)

AD is the most common dementia, characterized by extracellular amyloid-β (Aβ) plaques, intracellular tau tangles, and progressive cognitive decline [122, 123]. Over the past decade, multiple studies have revealed that AD patients harbor an altered gut microbiome compared to age-matched cognitively normal individuals [122]. A consistent finding is reduced overall microbial diversity in AD, along with a phylum-level shift: the proportion of Firmicutes (typically beneficial fiber-degrading bacteria) tends to be decreased, while Bacteroidetes are increased [124]. Levels of anti-inflammatory genera such as *Faecalibacterium* and *Eubacterium rectale* are often lower in AD, whereas certain pro-inflammatory or opportunistic taxa (like *Escherichia/Shigella*) are enriched [125, 126]. For example, one study found that cognitively impaired elderly with brain amyloidosis had *Escherichia/Shigella* overabundance and depleted *E. rectale*; notably, those changes correlated with higher peripheral inflammation (plasma cytokines) [127, 128]. This suggests a link between gut dysbiosis, systemic inflammation, and AD pathology. Indeed, neuroinflammation is a prominent feature of AD, and as discussed, translocated gut microbial products (e.g. LPS) have been detected at higher levels in AD patient brains and are known to activate microglia.

Mechanistically, several MGBA pathways appear to be involved in AD. In terms of immune modulation, AD patients often show peripheral immune abnormalities that could originate in the gut [129]. There is evidence of increased gut permeability in AD, which might allow more pro-inflammatory molecules to circulate [127]. SCFA deficits might also play a role: fecal levels of butyrate and other SCFAs are reported to be reduced in AD patients, which could exacerbate neuroinflammation by depriving microglia of anti-inflammatory signals [130]. Supporting this, germ-free AD model mice (which lack SCFAs and other microbial signals) have impaired microglial maturation and reduced plaque clearance, leading to greater amyloid accumulation [131, 132]. Recolonization of these mice with a complex microbiota (especially if it includes SCFA producers) partially restores microglial function and reduces Aβ burden [133]. Another study showed that antibiotics that drastically alter the gut microbiome can modulate amyloidosis: short-term antibiotic treatment in an AD mouse model altered gut bacteria and resulted in reduced plaque deposition and lower neuroinflammation [134]. However, only certain combinations of antibiotics had this effect, implying that specific microbial communities or functions are pathogenic, whereas others may be protective.

Metabolic pathways are also relevant in AD. The gut microbiota influences levels of bile acids, and AD patients have altered bile acid profiles in serum and cerebrospinal fluid (with higher ratios of toxic vs. neuroprotective bile acids) [135]. This might be due to microbial changes; some gut bacteria convert primary bile acids into secondary forms that can cross into the brain and activate receptors like TGR5 on glia, affecting inflammation and glucose metabolism in the brain [135]. A recent multi-omics study of AD patients identified a network connecting gut microbiome changes to fecal metabolites to brain imaging markers [136]. Notably, imbalances in microbial metabolites such as imidazole propionate and γ-aminobutyric acid (GABA) were linked to reduced brain glucose metabolism and cortical thinning in AD [137]. This suggests that gut-derived metabolites could contribute to the energy deficits and synaptic dysfunction observed in AD.

Another intriguing MGBA aspect in AD is the direct effect of gut microbes on amyloid and tau pathology. Some gut bacterial metabolites can interfere with Aβ aggregation [138]. For instance, certain microbial polyphenol metabolites inhibit Aβ fibrillization in vitro [139]. Conversely, *E. coli* producing the Curli amyloid exacerbated Aβ deposition in one mouse study [91]. Additionally, chronic infection or dysbiosis might drive peripheral inflammation that reduces the clearance of Aβ from the brain via the glymphatic system and BBB transporters [140]. There is also emerging evidence that the gut microbiome can affect tau pathology, possibly through inflammation-mediated kinase activation (microbial LPS and cytokines can activate kinases that phosphorylate tau) [140].

From a translational perspective, these findings raise the possibility of microbiome-based biomarkers and therapies in AD. Some have proposed that specific microbial taxa or metabolite profiles in stool could serve as early indicators of AD risk or progression [141]. For example, a high abundance of pro-inflammatory bacteria (like *Escherichia*) coupled with low SCFA producers might predict faster cognitive decline [130]. Therapeutically, small trials in humans are underway: one randomized trial in mild AD is testing an oral broad-spectrum antibiotic followed by FMT to “reset” the microbiome [142]. In animal models, similar approaches have shown that repopulating the gut with a youthful or diverse microbiome can improve cognitive function [143]. Probiotics have also shown promise (discussed further in the Therapeutic section). In one placebo-controlled trial, AD patients who received a daily multi-strain probiotic for 12 weeks had significantly better Mini-Mental State Exam scores and lower blood inflammatory markers than those on placebo [144]. While these improvements were modest, they demonstrate that manipulating the gut can impact inflammation and cognition in AD.

In summary, AD is accompanied by a distinct gut microbiome signature that likely contributes to disease via increased inflammation, reduced neuroprotective metabolites, and possibly direct effects on protein pathology. Therapies aimed at restoring a healthy microbiome or blocking deleterious MGBA signals (like LPS or certain bile acids) are being explored as novel ways to slow AD progression.

### Parkinson’s Disease (PD)

PD is a movement disorder marked by loss of dopaminergic neurons in the midbrain and accumulation of α-synuclein aggregates (Lewy bodies) [145, 146]. Gastrointestinal dysfunction is an early and common feature of PD – up to 80% of PD patients experience chronic constipation and other GI issues, often years before motor symptoms [147]. This prodromal phase, along with Braak’s hypothesis of an ascending gut-to-brain spread of α-synuclein, has drawn intense interest to the MGBA in PD [148, 149]. Numerous studies have now characterized the PD gut microbiome, consistently finding dysbiosis relative to neurologically normal controls [150]. A hallmark is the depletion of bacteria that produce SCFAs and support mucosal health. For instance, members of the *Prevotellaceae* family (such as *Prevotella* genus) are significantly reduced in many PD cohorts [151, 152]. *Prevotella* are fiber-fermenters that produce butyrate and also contribute to mucin synthesis in the gut; their paucity in PD may lead to less SCFA availability and a thinner protective mucus layer. Indeed, low fecal SCFA levels have been documented in PD, which could compromise gut barrier integrity and immune regulation [153]. In parallel, PD microbiomes often show an overrepresentation of certain opportunistic or pro-inflammatory microbes [154]. *Akkermansia muciniphila*, a mucin-degrading bacterium, is frequently enriched in PD stool samples [155]. While *Akkermansia* is often considered a beneficial microbe in metabolic contexts, in PD its overgrowth might reflect (or contribute to) excessive mucin erosion and gut barrier dysfunction [156]. Increased *Enterobacteriaceae* (a family that includes endotoxin-producing Gram-negatives) has also been reported and was correlated with the severity of postural instability and gait difficulty in one study [157]. In short, the PD gut microbiome tends to harbor fewer “good” SCFA-producing, anti-inflammatory bugs and more “bad” pro-inflammatory, mucus-depleting bugs.

How might these changes influence PD pathogenesis? One major pathway is neuroinflammation. Postmortem and Cerebrospinal Fluid (CSF) studies show PD has an inflammatory component, with activated microglia and elevated cytokines [158, 159]. Gut-derived LPS or peptidoglycans could be driving this if the intestinal barrier is compromised. Elevated intestinal permeability has been observed in PD patients, along with markers of endotoxemia in the blood [160]. The MGBA immune links described earlier (Th17 cells, etc.) are pertinent too – PD patients have been found to have increased Th17 cells in circulation, and recent research implicates gut bacteria in shaping this PD-specific immune profile [161]. For example, *Segmented Filamentous Bacteria* (SFB) in the gut potently induce Th17 cells; if PD dysbiosis includes SFB or others with similar effects, it could promote CNS inflammation that accelerates neurodegeneration [162]. Conversely, a lack of SCFA-producing *Roseburia* and *Faecalibacterium* (often reduced in PD) means fewer Tregs to keep inflammation in check [163].

Another key link is the vagal route of α-synuclein transport. As noted, α-syn pathology in PD might start in the gut (possibly triggered by a pathogen or toxin) and spread via the vagus nerve. Supporting this, α-syn aggregates have been identified in the enteric nervous system and vagus of early PD patients [164]. If the gut microbiome is altered, it might influence this process. For example, certain microbial metabolites (like SCFAs) can promote α-syn aggregation in enteric neurons, as shown in one mouse study [95]. Additionally, dysbiosis-induced intestinal inflammation could increase local α-syn expression (since α-synuclein is expressed in enteric neurons and is upregulated by inflammation) [165]. Once misfolded α-syn is present in the gut, it could propagate to the CNS more readily if vagal trafficking is enhanced by gut inflammation or hyperactivity of the ENS [166]. Epidemiologically, full truncal vagotomy (cutting vagus connections to gut) has been associated with lower PD incidence, hinting that in some patients the gut-to-brain route is critical [33].

Metabolic and endocrine factors are also at play. Constipation and slow transit in PD alter the fermentation patterns in the colon, potentially leading to increased production of metabolites like TMAO (from protein fermentation) which may aggravate neuroinflammation [167]. The microbiome can influence drug metabolism relevant to PD as well – a striking example is levodopa, the primary PD medication. Certain gut bacteria (e.g. *Enterococcus faecalis*) possess an enzyme that decarboxylates levodopa in the intestine before it can be absorbed, effectively reducing the drug’s availability [168, 169]. A 2025 study discovered this bacterial enzyme pathway and even identified an inhibitor that could block it [170, 171]. This finding means that differences in gut microbiome might contribute to the notorious variability in patient response to levodopa; it also suggests a possible therapeutic angle (pairing Parkinson’s meds with microbiome-targeted adjuvants to improve efficacy).

On the flip side, PD therapies and diet can affect the microbiome, creating feedback loops. For instance, some PD patients take amine oxidase inhibitors or anticholinergics that alter gut motility and bacterial growth [172]. Many PD patients also consume high-protein diets (to avoid losing muscle), which can shift the microbiome toward more proteolytic species (increasing potentially harmful metabolites like p-cresol and phenols) [173]. Investigations are underway to see if dietary interventions (like ketogenic or Mediterranean diets) can beneficially remodel the PD microbiome. A pilot ketogenic diet trial in PD suggested possible motor improvement, which might be partially due to changes in gut bacteria and their metabolites (ketone bodies can influence gut microbial composition) [174]. However, such extreme diets are hard to maintain, so more moderate dietary approaches are being studied.

From a clinical trial perspective [175], multiple microbiota-targeted interventions are being tested in PD. Randomized trials of various probiotic formulations have shown improvements mainly in gastrointestinal symptoms (e.g. reduced constipation, bloating) and some modest benefits in motor scores [176]. A recent meta-analysis concluded that probiotics significantly improve bowel movement frequency in PD and may provide a slight improvement in Unified Parkinson’s Disease Rating Scale (UPDRS) motor scores [177]. FMT has also moved into clinical trials for PD: a Phase II placebo-controlled trial (single nasojejunal infusion of donor stool) in early PD reported a mild but Statistically Significant improvement in motor symptoms at 12 months compared to sham transplant. Specifically, treated patients improved by ~ 5.8 points on the UPDRS-III (motor) versus ~ 2.7 points in controls, with benefits sustained for at least a year [175]. This suggests that altering the PD gut microbiome can indeed translate into clinical benefit, albeit modest. Ongoing studies are examining repetitive FMT dosing and different delivery routes. Other approaches include antibiotics like rifaximin (a non-absorbed antibiotic) to reduce overgrowth of potentially harmful bacteria; a small open-label trial of rifaximin showed some improvement in PD motor function and gut symptoms, but long-term use is not practical due to antibiotic resistance and microbiome disruption [178].

In summary, PD provides a clear example of a neurodegenerative disease wherein the MGBA is intimately involved. Gut microbiota changes in PD can contribute to α-syn pathology propagation, modulate neuroinflammation, influence drug metabolism, and exacerbate autonomic symptoms. Conversely, interventions to rebalance the microbiome hold potential to alleviate both motor and non-motor PD manifestations. Future research in PD is increasingly focused on identifying specific microbial metabolites or strains that could be targeted to slow neurodegeneration (for instance, boosting SCFA producers or inhibiting bacterial enzymes that interfere with host molecules). PD, perhaps more than any other NDD, exemplifies the concept that neurological diseases are not restricted to the brain but are truly systemic disorders involving the gut-brain axis.

### Multiple Sclerosis (MS)

MS is an immune-mediated disease characterized by autoreactive inflammation, demyelination of CNS axons, and progressive neurodegeneration [179]. It has features of both an autoimmune disorder and a neurodegenerative condition, which makes it a particularly interesting case for the MGBA [180]. In fact, MS was one of the first CNS diseases where gut bacteria were shown to have a causal influence in animal models: in 2011, it was demonstrated that *segmented filamentous bacteria* in the gut could trigger CNS-autoreactive Th17 cells and provoke an MS-like disease in mice [181]. Since then, multiple studies have found that the microbiome of MS patients differs from that of healthy individuals in ways that could promote inflammation [182]. Commonly reported alterations include a reduction in butyrate-producing bacteria such as *Faecalibacterium prausnitzii* and *Butyricicoccus*, and an increase in taxa that can induce pro-inflammatory responses (like *Akkermansia muciniphila*, *Prevotella* spp. in some studies, or *Methanobrevibacter* and *Eggerthella* in others) [183]. One study found that MS patients had higher levels of *Akkermansia* and *Acinetobacter* and lower *Parabacteroides* compared to controls, and when these microbial communities were transferred to germ-free mice, the mice developed more severe EAE (the mouse model of MS) [184]. Conversely, colonization of mice with certain commensals from healthy human guts can protect against EAE [185]. For example, *Prevotella histicola*, a human gut commensal, was shown to suppress CNS autoimmunity in mice, increasing regulatory T cells and suppressing Th17 cells [186].

In people with MS, immunological profiles correlate with gut microbiome composition. A notable finding is the higher frequency of pro-inflammatory Th17 cells in the gut and blood of MS patients, which correlates with microbiota alterations [67]. Cosorich et al. (2017) observed that MS patients with active disease had an abundance of *Akkermansia* and *Ruminococcus* (which can erode the mucous barrier) and this was accompanied by elevated Th17 cells in the gut mucosa [67]. The implication is that certain bacteria promote a Th17-skewed response that can migrate to the CNS and attack myelin. Additionally, reduced levels of SCFA-producers in MS may lead to a deficit in SCFAs like butyrate and propionate that normally help maintain Treg cells [187]. Indeed, a recent clinical study demonstrated that giving oral propionate (a microbial metabolite) to MS patients increased their peripheral Treg counts and was associated with a lower annual relapse rate over the ensuing 3 years [188]. This indicates that augmenting the function of missing beneficial microbes can tip the immune balance toward regulation rather than autoimmunity.

Beyond T cells, the gut microbiome might influence B cells and antibody responses in MS as well. Some gut bacteria share antigens that mimic myelin proteins, potentially triggering cross-reactive antibodies (a concept known as molecular mimicry) [189]. There is some evidence of IgA and IgG antibodies against gut commensals being elevated in MS, which might reflect an aberrant immune surveillance of the gut microbiota that spills over to CNS-directed immunity [190]. Additionally, metabolites from gut bacteria can affect microglia in MS [191]. For instance, tryptophan metabolites acting on the aryl hydrocarbon receptor (AhR) are decreased in MS, and stimulating AhR in astrocytes and microglia has been shown to reduce CNS inflammation [192, 193]. Certain *Lactobacillus* strains produce AhR ligands; not surprisingly, *Lactobacillus* is often found at lower abundance in MS microbiomes, and giving probiotic *Lactobacilli* in EAE ameliorates disease partly via AhR activation in the gut and CNS [194, 195].

Gut barrier integrity is another factor: MS patients in remission versus flare have been noted to have differences in fecal microbiota that may impact gut permeability. During active disease, higher levels of *Eggerthella* (a genus associated with intestinal inflammation) have been found, which might loosen the gut barrier and allow more immune activation [184]. A “leaky” gut in MS could enable translocation of bacterial fragments that activate innate immunity (e.g. LPS activating microglia via TLR4 as earlier described) [196]. Some MS patients also have co-existing inflammatory bowel disease or irritable bowel syndrome at higher rates than the general population, hinting at shared genetic or environmental factors affecting gut inflammation [197].

From a therapeutic standpoint, there is excitement about microbiome modulation in MS. Dietary interventions rich in fermentable fiber have shown immunological benefits in MS: a high-fiber diet increased SCFA levels, expanded Tregs, and improved EAE severity [198]. Human data aligns with this – MS patients who adhere to a Mediterranean-style diet (high fiber and unsaturated fats) tend to have lower disability scores and less inflammatory markers, though confounding lifestyle factors exist [199]. Probiotic supplementation in MS has been tested in several small trials [200]. A 2023 meta-analysis of these trials concluded that probiotics (usually multi-strain combinations of *Lactobacillus* and *Bifidobacterium*) led to a significant reduction in pro-inflammatory cytokines (like TNF-α and IL-6) and a slight improvement in patients’ Expanded Disability Status Scale (EDSS) scores [201]. One representative RCT found that a 12-week probiotic regimen in MS decreased IL-17 and increased IL-10 levels, indicating a shift toward an anti-inflammatory profile [202]. FMT is also being explored: an open-label trial of FMT in MS demonstrated safety and hinted at some improvements in gut microbiome diversity and fatigue scores, and a placebo-controlled Phase I FMT trial in progressive MS is currently underway to assess impacts on MRI lesions and clinical outcomes [203, 204].

Because MS straddles the immune/degenerative divide, combining microbiome therapy with existing immunomodulatory drugs is an area of interest. One study gave a probiotic alongside an MS immunotherapy and reported an augmented expansion of Tregs compared to drug alone [205]. Another intriguing approach is using microbial metabolites as adjuncts: as mentioned, oral propionate supplementation led to fewer relapses and increased Tregs in a cohort of MS patients [206]. There are plans to test butyrate supplements as well, given preclinical evidence that butyrate reduces demyelination and enhances remyelination in the CNS. PB-TURSO (sodium phenylbutyrate + taurursodiol) Slowed AlSFRS-R decline in the phase 2/3 CENTAUR RCT (basis for 2022 FDA approval) but failed in the phase 3 PHOENIX trial and was voluntarily withdrawn from U.S./Canada in 2024; targets HDAC/ER-stress pathways rather than the microbiome perse [207]. In MS, evidence to date does not show that TUDCA monotherapy reduces brain atrophy; rather, higher baseline bile acid levels correlate with slower atrophy, and a small randomized TUDCA study established safety and biologic target engagement without demonstrable clinical or imaging efficacy [208].

In conclusion, MS is strongly influenced by gut microbiota, with evidence at the molecular, cellular, and clinical levels. The microbiome can drive autoimmunity (through Th17 cells, molecular mimicry, and pro-inflammatory metabolites) or conversely promote tolerance and tissue repair (through SCFAs, Tregs, and neuroprotective metabolites). MS thus exemplifies how an imbalance in the MGBA can contribute to both initiation and progression of a neurologic disease. Targeting the microbiota in MS holds dual promise: calming the aberrant immune attack and simultaneously fostering a more neuroprotective CNS environment. Early clinical studies are encouraging, but larger trials will be needed to determine if microbiome therapies can meaningfully alter the course of MS beyond the effects of standard immunomodulatory drugs.

### Amyotrophic Lateral Sclerosis (ALS)

ALS is a rapid and fatal neurodegenerative disease affecting motor neurons, leading to paralysis [209]. Unlike AD, PD, or MS, the role of the MGBA in ALS is only beginning to be understood, but recent research suggests the gut microbiome may influence ALS progression and possibly patients’ metabolic status [210]. Clinically, ALS patients often have hypermetabolism and GI symptoms like weight loss, which could both affect and be affected by gut microbes [211]. Fecal microbiome analyses have found dysbiosis in ALS, though findings are not entirely consistent across studies (perhaps due to different diets and progression rates in patients) [212]. Common observations include a reduction in certain beneficial genera (such as butyrate producers *Roseburia* and *Faecalibacterium*) and an increase in pro-inflammatory genera (like *Escherichia* or *Oscillospira* in some reports) [213]. One study reported that ALS patients had signs of intestinal inflammation and dysbiosis with a shift toward microbes that can induce oxidative stress and reduce gut barrier function [214].

Animal models of ALS have provided stronger evidence for MGBA involvement. In the SOD1^G93A^ transgenic mouse (a common ALS model), researchers observed that the mice develop an altered microbiome even before symptom onset [83]. Moreover, rendering these ALS mice germ-free or treating them with antibiotics significantly accelerated their motor neuron degeneration, implying that some aspect of the microbiome is beneficial in ALS [83]. A groundbreaking 2019 study demonstrated that colonizing ALS mice with *Akkermansia muciniphila* (a mucin-degrading bacterium usually considered pro-inflammatory in PD/MS contexts) actually ameliorated ALS progression in the mice [83]. The reason turned out to be metabolic: *Akkermansia* produces nicotinamide (vitamin B3) as a metabolite, and nicotinamide levels were low in the ALS mice (and in ALS patients) [215]. Nicotinamide supplementation improved motor neuron survival in the mice, suggesting that *Akkermansia* was beneficial by supplying this neuroprotective metabolite. This finding is striking because *Akkermansia* was mentioned as potentially harmful in PD/MS, yet here it had a protective effect – highlighting that the impact of a given microbe can vary greatly depending on disease context and metabolic needs.

Other commensals have been implicated in ALS models as well. For example, Butyrate-producing bacteria might be beneficial in ALS (butyrate has neuroprotective properties, as described) [216]. In one study, ALS mice given a butyrate-producing bacterial cocktail showed delayed symptom onset and reduced neuroinflammation [217]. Another study found that *Parabacteroides distasonis* and *Ruminococcus torques* were overabundant in ALS mice and appeared to have adverse effects, whereas *Akkermansia* stood out as beneficial [83]. This suggests that selectively augmenting or inhibiting certain microbes could change disease outcomes. The mechanisms likely involve immune modulation (microglia in ALS can adopt a neurodegenerative phenotype that might be restrained by microbial signals) and metabolic support (providing nutrients like nicotinamide or SCFAs to neurons and glia) [214, 218]. In fact, a recent study reported that the microbiome in ALS mice helps restrain pro-inflammatory microglia, which is opposite to what happens in AD models [219]. So in ALS, rather than driving pathology, the baseline microbiome might be trying to counteract it, and losing key microbes removes that brake on microglial activation.

Clinically, there are hints that dietary and microbiome interventions could help ALS patients. ALS patients who consume high-calorie, high-fat diets have been noted to survive longer on average, possibly because it combats weight loss and maybe alters the microbiome to a more energy-extracting configuration [220]. In a small trial, ALS patients on a hypercaloric diet had a slower functional decline than those on a normal diet. Such a diet often increases *Akkermansia* in the gut (since *Akkermansia* thrives on mucin when fiber is low and fats are high), which as mentioned might produce nicotinamide and other beneficial compounds [211]. Probiotic trials in ALS are still in early stages. An ongoing pilot study is testing a multi-strain probiotic in ALS to see if it can improve GI function or inflammation [221]. No efficacy results are available yet, but safety is expected to be fine as in other populations [222]. FMT is also being considered – at least one case report described an ALS patient getting FMT, and anecdotal notes suggested some transient improvement in gastrointestinal symptoms and possibly motor function, but rigorous data are lacking. A planned trial of FMT in ALS will primarily look at tolerability and microbiome engraftment [223].

It is worth noting that one of the recently approved ALS therapies (sodium phenylbutyrate + TUDCA, as mentioned earlier) highlights the intersection of microbiome-related metabolism and neurodegeneration [224]. Phenylbutyrate is an HDAC inhibitor (similar action to butyrate from microbes) and TUDCA is a bile acid that can modulate gut microbiota composition as well as reduce ER stress in neurons [225]. This combination was shown to slow ALS progression modestly in trials. While not explicitly a microbiome therapy, it underlines manipulating metabolites common to host–microbe metabolism can impact ALS.

In summary, research in ALS suggests the gut microbiota can influence the pace of neurodegeneration and the metabolic state of the host. In contrast to PD/MS, where certain bacteria exacerbate disease, ALS might be a case whereas enhancing specific microbial functions (like vitamin production) is key. Given ALS’s rapid course, any stabilizing effect from the microbiome could be significant. The field is young, but ALS patients might one day receive personalized microbiome-based adjuncts – for example, a consortium of bacteria tailored to produce neuroprotective metabolites or to reduce neurotoxic ones – as part of a broader therapeutic regimen. Much remains to be learned, especially how to maintain beneficial microbes in patients who often have difficulty eating and maintaining gut health due to their illness. Nonetheless, ALS underscores that even diseases without a clear immune component can be shaped by the gut microbiome through metabolic and glial-modulating pathways.

## Microbiota-targeted therapeutic strategies and clinical trials

**Fig. 4:**
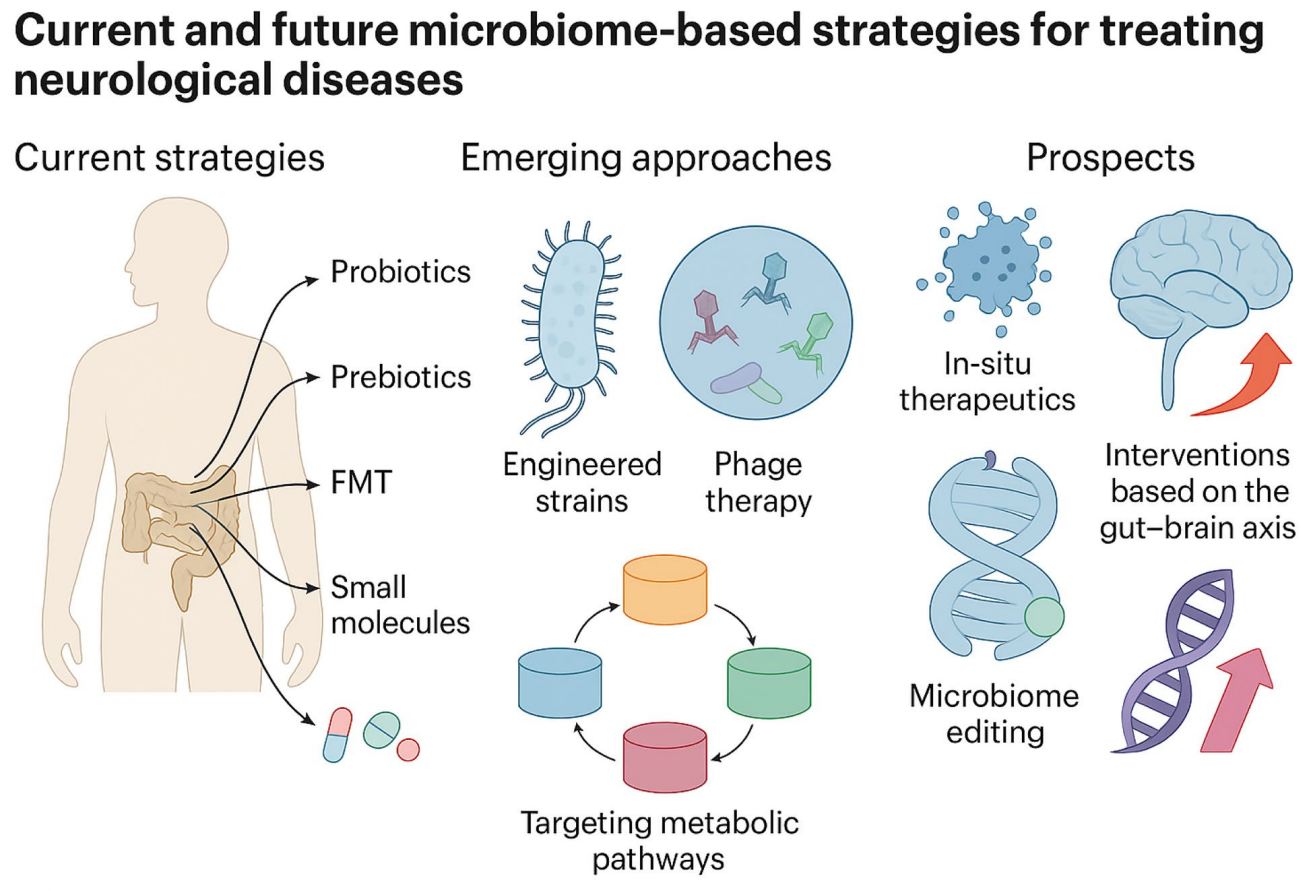
Therapeutic strategies targeting the MGBA. Interventions include probiotics, prebiotics, dietary regulation, fecal microbiota transplantation (FMT), bacteriophages, engineered bacterial strains, and metabolite-based therapies. The figure summarizes current approaches and their proposed mechanisms of action on central and peripheral MGBA components

Building upon mechanistic understanding, this section synthesizes interventional strategies targeting the MGBA. We cover dietary approaches, probiotics, FMT, and pharmacological agents, evaluating both their biological plausibility and clinical evidence. Given the evidence linking gut dysbiosis with neurodegenerative disease mechanisms, a variety of strategies are being pursued to modulate the microbiota–gut–brain axis for therapeutic benefit (Fig. 4). Broadly, interventions fall into a few categories: dietary modifications and prebiotics, probiotic supplementation, fecal microbiota transplantation, direct microbial metabolite (postbiotic) supplementation, and emerging approaches like phage or small-molecule therapies targeting microbial pathways. The goals of these strategies are typically to restore a healthy microbial community, enhance production of beneficial microbial metabolites, and/or reduce levels of pro-inflammatory or neurotoxic microbial products. Table 1 provides an overview of representative clinical trials testing MGBA-targeted interventions in neurodegenerative diseases.

**Table 1:** Representative clinical studies of MGBA-related interventions across neurodegenerative disorders

Neurological condition	Intervention	Design	n (analyzed)	Duration/follow-up	Primary outcome	Key secondary outcomes	Methodological notes	Safety/AE	Citation
Parkinson’s disease	Single-dose anaerobically prepared FMT via colonoscopy vs placebo	Double-blind, randomized, placebo-controlled (2:1) multicenter RCT (Finland)	45	6 months primary, 12 months follow-up	Change in MDS-UPDRS I–III (III off-med) at 6 months: no between-group difference	Placebo group showed greater improvement in some motor/non-motor measures and faster LEDD increase; microbiota shifts larger after FMT; dysbiosis reversal more frequent in placebo	Predefined primary endpoint; donor-dependent microbiota engraftment; screening n = 229; intention-to-treat n = 45	GI AEs higher with FMT (53% vs 7%)	[175]
Parkinson’s disease	Synbiotic (L. paracasei DG + inulin), open-label single-arm	Single-center clinical study	30	12 weeks	Non-motor symptom improvement (e.g., MDS-UPDRS I, SCOPA-AUT) from baseline	Constipation indices improved; *Faecalibacterium prausnitzii*; SCFA changes	No control arm; exploratory microbiome profiling in subset	No serious AEs reported	[226]
Parkinson’s disease with constipation	Mediterranean diet (dietary counseling) + standard care vs standard care	Parallel-group randomized controlled trial	36	8 weeks	Constipation symptom score improved with Mediterranean diet vs control	Fecal calprotectin; changes in fecal zonulin and diet quality	Calprotectin used as intestinal inflammation marker	Not highlighted; diet well tolerated	[227]
Amyotrophic lateral sclerosis (sporadic)	FMT (healthy donor) vs sham (saline + coloring)	Double-blind, randomized, placebo-controlled RCT	27	35 weeks total (visits at baseline, wk15, wk23, wk35)	ALSFRS-R change: no difference FMT vs placebo	No differences in respiratory function, strength, QoL, NFL; improvements in constipation and mood in FMT group	Early termination underpowered; microbiome shift with Bifidobacterium	GI AEs most common; no serious AEs	[223]
At-risk/older adults without dementia (US POINTER)	Multidomain lifestyle (physical activity, MIND diet, cognitive training, vascular risk mgmt) vs health education control	Community-based, cluster-randomized pragmatic trial (U.S.)	2111	2 years	No Significant difference in global cognition at 2 years (overall)	Benefits in specific cognitive domains in adherent subgroups; improvements in cardiometabolic risk factors	Baseline characteristics previously reported	Lifestyle interventions generally safe; AE not primary focus	[228]

This table summarizes high-quality and recent clinical investigations testing microbiota–gut–brain axis (MGBA)–related strategies in Parkinson’s disease (PD), Alzheimer’s disease/mild cognitive impairment (AD/MCI), multiple sclerosis (MS), and amyotrophic lateral sclerosis (ALS). Columns report the neurological condition, intervention and comparator, trial design, analyzed sample size (n), treatment duration/follow-up, prespecified primary outcome, key secondary outcomes, methodological notes (e.g., donor effects, adherence, underpowering), safety/adverse events, and a verifiable citation. Studies are ordered by disorder and, within each disorder, by level of evidence (randomized trials before non-randomized/open-label). Outcomes are transcribed as reported by the original publications without re-analysis. Negative or neutral primary endpoints are explicitly indicated to avoid outcome bias. Citations in the last column correspond to peer-reviewed sources suitable for verification

### Dietary interventions

Consistent with the taxonomy outlined in Sect. 5.1, we first consider dietary modulation as the foundational, patient-centered lever for long-term ecosystem steering. Diet establishes the ecological “set point” on which targeted microbial therapeutics can engraft and persist; Sects. 5.3.2 and 5.3.3 evaluate probiotics/prebiotics and FMT within this context. Diet is a primary shaper of the gut microbiome and a feasible, patient-centered therapeutic lever. Plant-forward patterns rich in fermentable fiber and polyphenols (e.g., Mediterranean or plant-based diets) are associated with a more eubiotic microbiome, increased production of short-chain fatty acids (SCFAs), and improved gut-barrier integrity. Epidemiological studies link higher adherence to Mediterranean-type diets with lower risk of Alzheimer’s disease (AD) and Parkinson’s disease (PD) and with slower cognitive decline [229–231]. These patterns emphasize fruits, vegetables, whole grains, legumes, and fish—sources of fiber and polyphenols that gut bacteria convert into anti-inflammatory metabolites [232, 233]. Despite adherence challenges, clinical studies report encouraging signals; for example, a modified Mediterranean-type intervention in mild cognitive impairment (MCI) increased microbiome diversity and reduced inflammatory markers [232, 234, 235]. In PD, a ketogenic diet trial (very high fat, low carbohydrate) is underway, hypothesizing shifts in the microbiome/metabolome (elevated ketones and possibly *Akkermansia*) with potential motor benefits [236].

In practice, dietary modification functions as a foundational, low-risk therapy that complements targeted microbial therapeutics: it can create a gut environment more receptive to colonization by probiotics/prebiotics and to sustained engraftment after FMT (see Sects. 5.2.2 and 5.2.3). Many clinicians advocate fiber-rich, omega-3-containing patterns such as the MIND diet—a Mediterranean/DASH hybrid tailored for brain health—which may simultaneously modulate the microbiome, provide neuroprotective nutrients, and reduce vascular risk factors relevant to neurodegeneration [237, 238]. Practical considerations include gradual fiber titration to minimize gastrointestinal symptoms and individualized support to improve adherence.

### Microbial therapeutics

Microbiota-targeted therapeutic strategies can be organized into a pragmatic taxonomy spanning (i) dietary modulation (addressed in Sect. 5.1), (ii) microbial therapeutics—namely targeted enrichment with probiotics and prebiotics versus community-level replacement via fecal microbiota transplantation (FMT) (Sect. 5.2), (iii) microbiota-derived/directed agents (“postbiotics”) such as short-chain fatty acids (SCFAs) and bile-acid modulators (Sect. 5.3), and (iv) pharmacologic or phage-based targeting of microbial pathways (Sect. 5.4). Across these modalities, shared mechanistic axes include ecological niche competition and colonization resistance; remodeling of metabolite networks (SCFAs, secondary bile acids, indole derivatives) with downstream signaling through G-protein–coupled receptors, FXR/TGR5, and the aryl hydrocarbon receptor; reinforcement of epithelial and neurovascular barriers; enteroendocrine and vagal signaling; and recalibration of innate and adaptive immune tone (e.g., microglial activation states, Th17/Treg balance). This scaffold clarifies where modalities overlap mechanistically yet differ in scope, durability, and standardization, providing a basis for explicit comparison and rational combination. Table 2 summarizes the main classes of therapeutic approaches, their rationales, and current evidence.

**Table 2:** Therapeutic strategy classes and evidence strength for MGBA-directed or MGBA-modulating interventions

Study/Intervention	Gut-brain pathway(s)	Mechanistic readouts	Interpretation	Citation
FMT vs placebo in PD	Microbiota composition/engraftment; intestinal inflammation; dopaminergic medication needs	Shotgun/16S changes by donor; dysbiosis reversal more frequent in placebo; symptom scales (MDS-UPDRS, NMSS), LEDD	Single-dose colonic FMT altered microbiota but did not improve clinical outcomes vs placebo; donor and bowel prep may be critical variables	[175]
Synbiotic in PD	SCFA producers (*Faecalibacterium*), enteroendocrine and immune modulation	*F. prausnitzii*; changes in fecal organic acids; improved non-motor scales	Signals for symptom relief with microbiome shifts, but open-label design limits causal inference	[226]
Mediterranean diet in PD	Dietary fiber and SCFAs; barrier/inflammation (calprotectin, zonulin)	Fecal calprotectin; improved constipation scores	Dietary modulation reduced intestinal inflammation marker alongside symptom gains	[227]
FMT in ALS	Microbiota immune axis (*Bifidobacterium*; mood/constipation)	*Bifidobacterium* through 15 weeks; no change in ALSFRS-R vs placebo	Underpowered negative primary outcome; non-motor improvements warrant further study	[223]
U.S. POINTER RCT	Indirect gut–brain effects via higher fiber/physical activity; vascular/metabolic modulation; no microbiome assays	Global cognition Slope: 0.243 vs 0.213 SD/yr (Δ + 0.029 SD/yr; 95% CI 0.008–0.050; P = 0.008)	Both arms improved; structured yields modestly greater gains; absolute effect vs natural history unclear (no non-intervention control)	[239]

This table organizes the principal intervention classes evaluated in neurodegeneration—multidomain lifestyle/dietary patterns, probiotics/synbiotics, fecal microbiota transplantation (FMT), postbiotics/metabolite therapy (e.g., butyrate, TUDCA), and targeted microbial-host pathways (e.g., bile acids, tryptophan–kynurenine)—and maps each to putative gut–brain pathways, mechanistic readouts, an evidence-based interpretation, and a concise evidence grade. Evidence grade reflects the highest-quality human data available for the entry (Level 1: randomized controlled trials; Level 2: non-randomized or Single-arm clinical Studies; Level 3: observational or mechanistic association; preclinical data considered supportive but not graded). Mechanistic readouts include stool/plasma metabolites (e.g., SCFAs), inflammatory and barrier markers (e.g., fecal calprotectin, zonulin, LBP/sCD14), medication needs (e.g., LEDD), and microbiome taxonomic/functional shifts. Safety and feasibility are interpreted from trial reports. This framework is intended to guide trial design (choice of endpoints, enrichment) rather than to rank efficacy across heterogeneous indications

#### Rationale and mechanisms

Targeted and replacement strategies act on overlapping axes—ecological niche competition and colonization resistance; metabolite reprogramming of SCFAs and secondary bile acids with signaling through GPCRs, FXR/TGR5, and AHR; reinforcement of epithelial and neurovascular barriers; and recalibration of innate and adaptive immune tone—yet differ in breadth, controllability, and durability. Probiotics and prebiotics aim to enrich defined taxa or feed specific guilds to shift functions in a tractable, label-able manner, often contingent on baseline community context and diet. FMT introduces a complex donor consortium that can restore missing cross-feeding networks quickly but at the cost of donor dependence and greater procedural and regulatory complexity. These distinctions motivate different endpoints (e.g., strain persistence vs. donor engraftment), time-to-effect expectations, and opportunities for induction–maintenance sequencing.

#### Probiotics and prebiotics

Probiotics—typically well-characterized *Lactobacillus/Bifidobacterium* consortia or next-generation strains—and prebiotics such as inulin-type fructans, galacto-oligosaccharides, and resistant starches offer standardized, scalable levers to steer microbial functions and metabolite profiles [240, 241]. Across preclinical and early-phase human studies, signals include improved barrier integrity, attenuation of low-grade inflammation, and modulation of neuroimmune pathways; synbiotics and psychobiotics seek to enhance effect sizes by pairing strains with substrate specificity and targeting mood/cognition-relevant circuits [242–245]. Safety is generally favorable (transient gastrointestinal symptoms predominate), but benefits are heterogeneous, strain/context dependent, and colonization is often transient, underscoring the value of fiber-forward dietary backgrounds (Sect. 5.3), responder stratification, and trials that predefine mechanistic biomarkers and product characteristics (identity, potency, dose, viability) [242–247].

#### Fecal Microbiota Transplantation (FMT)

FMT delivers a processed donor consortium to reconstitute community structure and function and is established for recurrent *Clostridioides difficile* infection; in neurodegenerative and neuroinflammatory contexts it remains investigational [248–251]. Early case series and open-label studies support feasibility and biological plausibility, whereas randomized trials show mixed clinical signals, highlighting donor-dependent effects, protocol heterogeneity (preconditioning, route, dose/frequency), and the need for engraftment-aware endpoints [252–255]. Safety is dominated by transient gastrointestinal events, with rare but serious risks of pathogen or antimicrobial-resistant organism transmission necessitating rigorous donor screening, validated manufacturing, traceability, and post-procedure surveillance under regulated protocols [256, 257]. Overall, optimization of donor selection, dosing schedules, and diet co-interventions appears pivotal to demonstrate consistent benefit [258].

#### Comparative effectiveness and use cases

Modality selection should weigh indication and urgency of effect, comorbidities and medication burden, standardization and labeling requirements, scalability and access, and patient preference. FMT can produce larger, faster ecological shifts but entails greater operational and regulatory burden; probiotics/prebiotics integrate readily with lifestyle programs, suit prevention and maintenance, and are easier to standardize, albeit with modest and variable effect sizes [242–247, 256–258]. Hybrid induction–maintenance designs—e.g., short FMT induction followed by synbiotic maintenance—or diet-anchored step-up approaches may combine breadth with durability; comparative studies should prespecify shared mechanistic readouts and clinically meaningful outcomes.

#### Safety, standardization, and regulatory considerations

For targeted strategies, risk is low when products meet strain-level identity, potency-at-expiry, and contaminant specifications under GMP-aligned manufacturing; vigilance is warranted in severely immunocompromised hosts. FMT requires comprehensive donor screening (including MDROs), validated processing with chain-of-custody and retention samples, release criteria, and pharmacovigilance; outside *C. difficile* infection, most jurisdictions restrict use to regulated trials [256–258]. Across modalities, reproducibility is limited by heterogeneity in formulation, dose, viability, and reporting. Adoption of harmonized product characterization and core mechanistic panels (e.g., SCFAs, bile acids, barrier/immune biomarkers) would materially improve evidence synthesis and regulatory appraisal.

#### Future directions

Next-generation approaches aim to capture community-level benefits with pharmaceutical-grade consistency: defined consortia (“FMT in a pill”) and engineered live biotherapeutic products with tunable functions and biocontainment. Precision will be enhanced by baseline phenotype–guided stratification (microbiome, metabolome, immune signatures), diet–microbe matching, and digital adherence support. Trial designs that implement induction–maintenance sequences, co-primary mechanistic and clinical endpoints, longer follow-up for durability, and head-to-head comparisons under harmonized outcome sets are priorities; diet co-interventions (Sect. 5.3) remain pragmatic tools to enhance engraftment and sustain functional gains across modalities.

### Microbial metabolite (postbiotic) supplementation

Rather than delivering microbes, an alternative approach is to deliver beneficial microbial products (or “postbiotics”) directly [259]. This can ensure a controlled dosage and avoid uncertainties of live microbial behavior. Examples include SCFAs like butyrate or propionate, which can be given orally or even intravenously [260]. Sodium butyrate, as discussed, has shown neuroprotective effects in multiple mouse models (AD, PD, MS) [76]. In an MS model, butyrate administration led to increased remyelination of neurons [261]. In AD models, it improved memory even when given late, presumably by enhancing histone acetylation and BDNF expression [262]. Small human studies are now starting: one trial in MS is testing oral sodium butyrate’s effect on MRI and immune markers [263]. Propionate, another SCFA, has already shown an impact in MS patients, as noted (higher Tregs and reduced relapses) [264]. Outside of SCFAs, other postbiotics of interest include tryptophan metabolites. For instance, indole-3-propionic acid (IPA) is a microbial metabolite with antioxidant properties that can cross the BBB; low IPA levels have been associated with worse cognitive function [265]. Efforts are underway to formulate IPA or similar compounds as supplements. Another category is vitamins and cofactors – since gut bacteria produce vitamins B6, B8, B12, K, etc., supplementing these might compensate for dysbiosis [266]. Vitamin B3 (nicotinamide) was effective in ALS mice via *Akkermansia*, and now nicotinamide is being trialed in ALS patients at high doses to see if it slows progression [83, 267]. Similarly, urolithin A (a microbial metabolite from polyphenols) is being explored for its mitophagy-boosting effects that could benefit aging neurons [268, 269]. The challenge with postbiotics is ensuring they reach relevant tissues in active form and determining optimal dosages (levels of these compounds in a healthy person’s gut can be quite high locally, which is hard to replicate with oral dosing due to absorption and metabolism). Nonetheless, they represent a precision approach – pinpointing which molecular deficiencies exist due to dysbiosis and correcting them. One successful example from another field is oral bile acid supplements for certain neurological disorders; in some ataxias, adding a bile acid (like TUDCA) thought to be deficient due to microbiome issues has improved clinical outcomes [270]. In NDDs, trials with TUDCA in ALS have shown some benefits (TUDCA is now approved in ALS as part of the PB/TUDCA combo) [224, 225]. Thus, directly supplementing key microbial metabolites – SCFAs, vitamins, amino acid derivatives, bile acids – is a promising adjunct or alternative to live microbes, especially for patients who might be immunocompromised or unable to tolerate FMT/probiotics.

### Targeting microbial pathways with drugs or phages

A nascent but exciting area is using targeted therapies to modulate the microbiome without necessarily adding anything new. One approach is selective inhibition of harmful microbial enzymes. The case of levodopa degradation in PD is illustrative: scientists identified a gut bacterial tyrosine decarboxylase that was eating up patients’ Parkinson’s medication, and they found a small-molecule inhibitor that could block this enzyme and thus prevent levodopa loss. This kind of approach – akin to an antibiotic but ultra-narrow in spectrum – could be used for other microbial pathways too. For example, inhibitors of TMA-lyase (the microbial enzyme turning choline into TMA) have been developed (e.g. DMB, 3,3-dimethyl-1-butanol) to reduce TMAO levels and are being tested in models of cardiovascular diseases [271, 272]. If a subset of gut microbes in AD or VD (vascular dementia) is driving TMAO-associated inflammation, such inhibitors might ameliorate it [273, 274]. Another target could be microbial proteases or toxins that degrade the mucus barrier or trigger inflammation; drugs that neutralize these could protect the gut lining. In parallel, bacteriophage therapy is being considered – phages are viruses that selectively infect bacteria [275, 276]. One could, in theory, use phages to knock down specific pathogenic bacteria in the gut without affecting the rest (unlike broad antibiotics). There is research into phages that target *Enterobacteriaceae* or *Proteobacteria* overgrowth, which could be relevant for PD where those are elevated [277]. Engineering phages to deliver genes into gut bacteria is another futuristic approach, potentially turning a microbe harmful to harmless. Additionally, some existing drugs not originally aimed at the microbiome turn out to have microbiome-mediated effects. Metformin, a diabetes drug, alters the gut microbiome and increases SCFAs and bile acids that activate GLP-1, which has led to exploration of metformin in AD and PD trials for its possible MGBA benefits [278, 279]. Minocycline, an antibiotic that crosses the BBB, has anti-inflammatory effects in the brain and also changes the gut microbiome composition in anxiety models [280–282]. While not a targeted microbiome drug, its pleiotropic effects (part immune modulation, part microbe modulation) have been tested in early PD and HD trials (with mixed results). The bottom line is that the pharmacologic toolkit for modifying the microbiome is growing. We may eventually see combination therapies – e.g. a patient gets an FMT or probiotic to establish a core healthy microbiome, then a targeted enzyme inhibitor to prevent that microbiome from producing a particular neurotoxin, plus a diet to feed it the right substrates. This multipronged strategy recognizes the complexity of the MGBA and the need to tackle it at multiple levels for maximal therapeutic effect.

It is important to note that not all trials of microbiota-targeted therapies have been successful. There have been instances of null results – for example, a recent trial of *Synbiotics* (combined prebiotic + probiotic) in PD did not show significant motor benefits, possibly due to insufficient dosing or variability in patients’ baseline microbiomes [283]. Similarly, an antibiotic trial in AD aimed at altering the microbiome did not yield cognitive improvement over placebo [284, 285]. These outcomes highlight challenges such as inter-individual differences (what works in one person’s gut may not in another’s), timing (intervening too late in disease may limit benefits), and the need for biomarkers to identify who is most likely to respond. As we move forward, personalizing microbiome therapies will likely be necessary – for instance, stratifying patients by their microbiome profile or metabolite levels, then tailoring the intervention (one patient might need more SCFAs, another needs more anti-TMAO measures, etc.).

Another challenge is ensuring long-term engraftment: probiotics often only transiently colonize, and FMT engraftment can wane with time if not maintained by diet or repeat dosing. Therefore, sustained lifestyle changes or periodic “boosters” might be required for durable benefits. Safety monitoring is also crucial – while probiotics and FMT have been safe in trials so far, there is a theoretical risk of introducing infections or causing undesired immune reactions, especially in patients with immune dysfunction (like those on MS immunosuppressants). Regulatory oversight will require standardized manufacturing for any live biotherapeutic products.

Despite these hurdles, the overall trend in clinical research is optimistic. Early-phase trials indicate that targeting the MGBA is feasible and can yield symptomatic improvements. Translationally, we also see that MGBA research is informing biomarker development: for example, measuring short-chain fatty acid levels, gut permeability markers, or fecal bacterial gene profiles as surrogate markers of treatment response in trials. In PD, researchers are testing whether a shift in stool microbiome after an intervention correlates with motor changes, which could validate that the therapy engaged the intended target (the microbiome).

In conclusion, a suite of microbiota-targeted strategies – diet, pre/probiotics, FMT, postbiotics, and precision drugs – are under active investigation as novel treatments for neurodegenerative diseases. These approaches are fundamentally different from traditional CNS drugs, as they work holistically through the gut-brain axis, potentially influencing multiple pathways (immune, metabolic, neural) simultaneously. While still in early days, they offer a complementary avenue to directly CNS-targeted therapies like monoclonal antibodies or neurotrophic drugs. Ultimately, the best care for NDDs might involve combining both: for example, using a disease-modifying drug to reduce protein aggregation in the brain and a microbiome therapy to reduce inflammation and enhance resilience. This multi-targeted approach, embracing the MGBA, aligns with the emerging view of neurodegenerative diseases as whole-body disorders that require system-level interventions.

In implementing these interventions, it’s likely that combinations will yield the best outcomes. For example, in an AD patient one might use diet to lay the groundwork, a probiotic to introduce a specific function (like producing more butyrate), and a small-molecule inhibitor to suppress a detrimental metabolite (like TMAO) simultaneously. Each strategy has distinct strengths – diet and probiotics broadly improve the microbiome’s balance and metabolites; FMT can reset a severely dysbiotic system; postbiotics and drugs can fine-tune specific pathways. An integrated approach, possibly personalized to each patient’s microbiome profile, will probably be required to significantly modify disease course.

Taken together, MGBA-targeted therapies have shown promise in modulating disease symptoms and biomarkers, yet most studies remain preliminary. Standardization of protocols, patient stratification, and integration with disease-modifying therapies will be critical for translation into clinical practice.

## Conclusion and future perspectives

Research into the microbiota–gut–brain axis has uncovered a vital new dimension in our understanding of neurodegenerative diseases. The gut microbiome, through its dynamic interactions with the immune, metabolic, and neural systems, emerges as both a contributor to pathology and a promising therapeutic target. In conditions like AD and PD, we now recognize that brain disorders are not “all in the head” – they involve a constellation of systemic changes, with gut dysbiosis potentially seeding inflammation and protein misfolding long before neurons degenerate. In MS, the microbiome’s influence on autoimmunity underscores the importance of environmental factors in neuroinflammation. Even for ALS, traditionally viewed as a cell-autonomous neurodegeneration, the surprising benefits of certain microbes hint at new metabolic avenues for intervention.

From a molecular viewpoint, key mechanistic themes have emerged: microbial metabolites such as SCFAs, tryptophan metabolites, and bile acids can penetrate into the CNS or signal via the vagus, modulating microglial activation, astrocytic support, and even neuronal gene expression. Conversely, microbial toxins like LPS or excessive ammonia can exacerbate neuroinflammation and oxidative stress. The balance of these influences may determine whether the CNS environment tilts toward neurodegeneration or repair. The MGBA thus offers a *multimodal* therapeutic target: unlike a drug that hits one receptor, manipulating the gut can simultaneously affect numerous pathways (immune, metabolic, etc.) that are dysregulated in NDDs. This systems-level modulation is both an opportunity (for comprehensive disease modification) and a challenge (for precision and predictability).

Clinically, the early trials give reason for cautious optimism. While not a panacea, microbiota-based interventions have shown the ability to improve at least some outcomes (e.g. cognitive scores in AD, motor symptoms in PD, inflammatory markers in MS). Importantly, many of these therapies are low risk and cost-effective – for instance, probiotics and dietary changes can be implemented alongside standard treatments with minimal downside. The variability in trial results also teaches us that a one-size-fits-all approach is unlikely to work. Patients have unique microbiome “fingerprints,” so future efforts must focus on personalized microbiome medicine. This could mean using baseline stool analyses to guide therapy selection – for example, a PD patient lacking SCFA-producers might benefit most from a butyrate-producing probiotic, whereas one with high Enterobacteriaceae might need a targeted phage or antibiotic to reduce endotoxin load.

Another critical area is the search for biomarkers and surrogate endpoints related to the MGBA. These would greatly enhance clinical trials and patient monitoring. Possibilities include: fecal SCFA levels (as an indicator of beneficial fermentation), serum TMAO (marker of detrimental microbial metabolism), gut permeability assays (like urinary sugar tests or blood zonulin) to gauge gut barrier integrity, and even gut microbial gene profiles that might predict rapid vs slow disease progression. For example, one could envision an “Alzheimer’s dysbiosis index” based on the ratio of certain bacterial taxa that correlates with cognitive decline rate – some studies are already exploring composite indices of this sort. Additionally, neuroimaging and CSF biomarkers can be integrated; one pioneering study linked gut metabolomic changes to functional brain MRI connectivity changes in AD. In PD, researchers are examining if gut microbiome composition associates with markers like alpha-synuclein in colonic biopsies or with REM sleep behavior disorder severity (a prodromal feature). The hope is that microbiome markers could serve as early warning signs (e.g. identifying high-risk individuals who could adopt preventative diets) or as indicators of therapeutic response (seeing the microbiome shift towards a “healthy” state might precede and predict clinical improvement).

As we look to the future, several directions are particularly exciting:

Longitudinal Cohort Studies: Following large cohorts over time with integrated gut microbiome and neurologic assessments will help nail down cause-effect relationships. If certain microbial changes consistently precede disease onset by years (as constipation does in PD), it strengthens the case for causality and prevention. Some such studies are underway (e.g. profiling microbiomes in people with genetic risk for PD or AD to see if and how their gut changes as they convert to disease).

Multi-omics and Systems Biology: Combining genomics, transcriptomics, metabolomics, and metagenomics will provide a holistic view of the host-microbe ecosystem in NDDs. For instance, single-cell RNA sequencing of microglia in germ-free vs colonized mice with neurodegenerative pathology can pinpoint microglial genes influenced by the microbiome. Similarly, metabolomic profiling of blood and CSF in patients, alongside microbiome data, can identify which microbial metabolites truly cross into the brain and affect pathways like amyloid deposition or axonal integrity. Machine learning on such complex datasets might reveal unexpected microbial influences (e.g. a bacterial product that correlates with tau phosphorylation levels). This systems approach will also help identify novel therapeutic targets – perhaps a particular microbial enzyme or pathway not previously linked to neurodegeneration could emerge.

Mechanistic Studies in Gnotobiotic Models: Animal models where the microbiome is controlled (germ-free or colonized with defined communities) are invaluable for proving mechanistic links. We have already seen examples: transplanting microbiomes from patients into mice to induce disease features, or adding/removing single species to gauge their impact on pathology. Continued use of these models, including newer ones like gut organoids with human microbiota or “brain-gut” chip systems, will allow dissection of how specific microbes or metabolites act on specific brain cell types. For example, one could test how *Akkermansia* nicotinamide affects motor neurons in vitro, or how a mix of SCFAs alters microglial gene expression in a co-culture system.

Therapeutic Development: Building on early successes, we anticipate second-generation therapies. Instead of crude FMT, maybe encapsulated defined consortia, with pharma-grade manufacturing, will be approved for NDD indications. Already, standardized FMT capsules are approved for *C. diff* infection – similar products might be tailored for AD (e.g. containing microbes that produce more butyrate and consume tryptophan into indoles). Probiotics might evolve into prescription “Live Biotherapeutic Products” with genetically enhanced functions – for instance, a *Bifidobacterium* engineered to overproduce glutathione (an antioxidant) to help with PD oxidative stress. Postbiotic drug development will likely expand; companies are examining compounds that mimic bacterial metabolites but are more drug-like (improved stability, BBB penetration). We may also see *adjunctive therapies* that combine microbiome modulation with neuroimmune modulation – e.g. pairing a gut-targeted therapy with an anti-amyloid or anti-synuclein antibody to tackle different aspects of disease.

Personalized and Preventive Approaches: In the more distant future, routine screening of one’s microbiome could become part of preventive neurology. If an individual in mid-life has a “pro-neurodegenerative” microbiome signature (low diversity, low SCFA-producers, high pro-inflammatory taxa), they might be counseled to intervene early via diet, prebiotics, or even prophylactic probiotics to shift their microbiome to a healthier state. Particularly for those with a family history or genetic predisposition (like APOE4 carriers for AD), microbiome-based prevention could be an attractive low-risk strategy to reduce risk or delay onset. This will require strong evidence from trials that such interventions in asymptomatic or early-stage individuals truly alter the trajectory – studies like the “Brain-Microbiome” project in prodromal AD and the “TopHat” trial in REM-sleep-behavior-disorder (prodromal PD) are beginning to explore this.

Challenges remain: Deciphering causality is difficult – the microbiome is both a cause and consequence of disease in many cases. We must avoid oversimplification; not all changes in the microbiome are harmful (some may be compensatory). There is also significant person-to-person variability – two AD patients might have very different microbiome alterations yet end up with similar pathology, indicating multiple microbial paths to the same disease. Thus, therapies will need to be adaptable or broad-acting. Regulatory aspects of microbiome therapies (especially FMT and genetically engineered microbes) require careful oversight to ensure safety and consistency. Additionally, patient acceptance is a factor – convincing patients to take say, an FMT capsule derived from stool, or to adhere to a strict diet, can be challenging; education and demonstrating clear benefits will help.

In conclusion, the microbiota–gut–brain axis provides a powerful lens through which to reinterpret neurodegenerative disease mechanisms, integrating neurology with immunology and metabolism. It shifts the paradigm from treating the brain in isolation to treating the “whole patient” – brain and body as an interconnected system. By harnessing this axis, we open up a new frontier of multi-targeted interventions that could complement existing therapies. The work ahead will determine how far we can go in translating MGBA science into tangible clinical benefits, but the progress to date already suggests that the gut microbiome may become a cornerstone of precision medicine in neurology. As this field advances, it brings hope for more effective, personalized, and holistic strategies to combat neurodegenerative diseases – transforming how we prevent and treat these formidable disorders that pose one of the greatest challenges to healthy aging in our society.

## Data Availability

Not applicable.

## Abbreviations

AD: Alzheimer’s disease
ASD: Autism spectrum disorder
BBB: Blood–brain barrier
BDNF: Brain-derived neurotrophic factor
CNS: Central nervous system
CRF: Corticotropin-releasing factor
EAE: Experimental autoimmune encephalomyelitis
ENS: Enteric nervous system
FMT: Fecal microbiota transplantation
GABA: Gamma-aminobutyric acid
GLP-1: Glucagon-like peptide-1
HD: Huntington’s disease
HPA axis: Hypothalamic–pituitary–adrenal axis
LPS: Lipopolysaccharide
MGBA: Microbiota–gut–brain axis
MS: Multiple sclerosis
PD: Parkinson’s disease
SCFAs: Short-chain fatty acids
TMA: Trimethylamine
TMAO: Trimethylamine N-oxide
TNF-α: Tumor necrosis factor-alpha
Th17: T helper 17 cells
Tregs: Regulatory T cells
